# Can the right to science redress inequitable access to innovative cancer therapy? A case for the justiciability of this ‘lesser known’ human right

**DOI:** 10.1093/jlb/lsaf017

**Published:** 2025-09-13

**Authors:** Ghada A Zakout

**Affiliations:** Erasmus University Rotterdam, Erasmus School of Law, Burg. Oudlaan 50, 3062 PA Rotterdam, Netherlands

**Keywords:** right to science, cancer research, cancer healthcare inequity

## Abstract

The human right to science, including the right to enjoy the benefits of scientific progress, is by far the least understood human right despite its central role in shaping scientific innovation. Research into innovative cancer therapies and biotechnologies has been pivotal in the realization of much of these advancements. Yet much of it is not accessible, affordable, or available to patients who need them most. This article examines the nature and scope of the right to science in cancer research in the context of General Comment No. 25 on Science and Economic, Social and Cultural Rights (E/C.12/GC/25) and Article 15 of the International Covenant on Economic, Social and Cultural Rights. The normative and ethical imperative of the right is annotated to provide a basis for its justiciability when redressing the pervasive issue of inequitable access to innovative cancer therapy. It argues that a constitutional dialogue that conceptualizes the right to science is warranted when rethinking ways ethical and human rights friendly research can be achieved. This renewed interest comes at a critical juncture when science in its contemporaneous situation needs to tackle cancer healthcare inequities amid turbulent geopolitical and epidemiologic challenges while addressing the rising cancer burden globally.

## I. INTRODUCTION

Our scientific understanding of cancer that lays down the foundations for the paradigm shift in the way cancer is diagnosed and managed has dramatically accelerated in the last few decades.^1^ It has led to significant transformational developments in the field of oncology, most notable for immunotherapy, targeted therapy, precision medicine, and the use of artificial intelligence and machine learning in research and clinical practice.^2^^,^^3^ This helped scientists and clinicians alike make life-changing ‘bench-work’ scientific cancer discoveries to ‘bed-side’ innovative cancer diagnostics and therapeutics.^4^ Cancer research is central to these developments. Access to new bio-scientific advances in cancer though remains a challenge to bringing equitable and affordable cancer drugs globally, particularly so in low- or middle-income countries (LMIC).^5^ There remains a significant gap where those who need it most are still disadvantaged and/or marginalized in participation, access and contribution to sciences, and in enjoying of their benefits as the dialogue seems to have shifted.^6^ It has long focused on two specific issues that are, for one, underscored by the advent of precision oncology and more recently perpetuated by the COVID-19 pandemic: (1) whether access to innovative, potentially life-saving, or curative cancer therapies only available on a research basis could be perceived as a constituent of right to science (RtS) and (2) whether and to what extent the normative 2020 General Comment No. 25 on Science and Economic, Social and Cultural Rights (E/C.12/GC/25)^7^ reflect today’s science enterprise that seems to strengthen intellectual property rights (IPR) protection and which hinders efforts to access to life-saving cancer treatments at reasonable costs.^8^ In that, the challenges for access to the benefits of cancer research appear refracted through geo-economic political agenda rather than legal.

From a legal perspective, flexibilities afforded by international trade agreements allow, under certain conditions, the production and sale of generic versions of anticancer agents that address germane cancer health needs in LMIC. But recent changes to European Union (EU) law on clinical research undermines this objective as it continues to focus on market optimization of the wider private-sector pharmaceutical development pipeline over public healthcare objectives^9^. Furthermore, the concept of the RtS has seldom been invoked in this international context with court decisions,^10^^,^^11^ hardly if any at all, explicitly acknowledging the RtS for access to innovative therapies.

Against this backdrop, I shall consider the legal and ethical implications of the Universal Declaration of Human Rights (UDHR)^12^ and the International Covenant on Economic, Social and Cultural Rights (ICESCR)^13^ in advancing the benefits of cancer research guaranteed by the RtS. I will outline a comprehensive and systematic overview of the normative basis and content of the RtS, dissect international jurisprudence based on a diverse range of sources, including international and regional (by which I chose Europe for its centrality of upholding human rights to its socio-political order, rule of law, and its emerging role as a global health actor^14^ in the current political climate^15^) biomedical research laws and policies, and bioethical frameworks and explicate the vulnerabilities of LMIC that are likely to be impacted by scientific advancements within the realm of implementing this fundamental right. And I conclude by calling for the channeling of the human rights imperative that affords a foundation for an equitable and just global health law as a means for challenging private and public inequities in global cancer healthcare.

## II. THE RTS AND HUMAN RIGHTS–A DEFINED NORMATIVE LEGITIMACY OR A CONCEPT OF CONVENIENCE?

Science is undoubtedly vital for the health and wellbeing of society. Its intrinsic values morally complements several humanitarian values including and not limited to integrity, rationality, precision, justice, objectivity, and impartiality that predicate its ethical standing. But despite being explicitly enshrined in existing international human rights law, this right has long been overlooked^16^ and even perceived as a privilege,^17^ so much so that some legal scholars referred to it as the ‘Cinderella of human rights’^18^ or ‘sleeping beauty’.^19^^,^^20^ So what is the RtS? What exactly is the scope of Article 15 and what is it protecting? Is it an inference that such a right should be considered as one of the human rights? Is it about the equitable distribution of the benefits of scientific insights?

Inspired by the French Revolution, Czech-French jurist Karel Vašák proposed the concept of the ‘three generations’ of human rights in 1979 at the International Institute of Human Rights in Strasbourg. Within this ontology, the RtS evolved as the second generation with the latter conceived as a movement that developed as a reprisal against capitalistic endeavors that puts equality before the law and individuals over States. It emphasized human rights standing in social, economic, and cultural aspects of human life. Classed under the nineteenth-century social-economic-cultural right, it was the by-product of the ‘abusive exploitation of the working class and of colonial people’ that unwillingly tolerated the uncritical conception of individual liberty.^21^ Thus, this right is seen as a ‘positive right’ in that it calls on states to actively intervene to ensure the equity of the values included in this right. It has dual potential to protect the value of science in society and ensure that science that serves humanity is worth discovering and appreciated as a framework to govern scientific progress; not merely a matter of privilege or generosity, rather a necessity.

Much like its inception, the RtS interpretation should be grounded in realizing its status as a cultural right so that it affords ingenuity and public dialogue in its future application. In doing so, it affords the opportunity for opening dialogue and collaboration between science actors and the public beyond a technocratic approach. This harnesses innovation and ensures that scientific advancements are not limited to their intended beneficial outcomes but transcend to being socially acceptable and ethically grounded. For one, addressing a fundamental moral debate of how it can be applied justly; ergo guiding the principles of distributive justice^22^ as a concept^23^ and not a conception. The RtS was initially conceived in the American Declaration of the Rights of Duties of Man^24^ and then negotiated in Article 27 of the UDHR, the first international legislation to recognize it. The UN emphasized and expanded this declaration by turning it into a legally binding obligation under international law in 1966 to include the need for protection and dissemination of scientific progress through Article 15(1)b of the ICESCR.^25^ Its interpretation purports the principles of the right to socially responsible science.^26^^,^^27^ It also encompassed the right to participation in scientific progress. Additionally, it incorporated the ‘freedom of the researchers’ as a requisite and collaboration in science (Article 15(3) ICESCR).^28^ Accordingly, the premise of Article 15 presents two layers to such right, one being the RtS is increasingly linked to several other declared human rights, which gained traction in its role in humanities such that the right to scientific progress is materialized on humanity’s terms—a product of both its time and a timeless way of showing how nations drew on knowledge and experience and worked together toward a common goal but also as a catalyst for fielding policy-making and advocacy of socially responsible science. The indivisible and interdependent nature of human rights, which was reaffirmed most recently in the Vilnius declaration,^29^ where no one human right overrides another, meant that science must be conducted to serve goals conducive of the wider human rights framework rather than being fragmented or siloed if its objectives are to be realized—an approach also posited by van Beers when addressing the human genome editing quandary in the CRISPR era.^30^ This added a legal and moral layer to benefitting from advancements in science and technology, access to data, materials, and information aimed at furthering science and human rights’ causes on the one hand and the right of protection of the scholarly interests of the results on the other. Another is that it poses a progressive obligation on governments and states to adopt measures that uphold the right to the benefits of scientific progress^31^—a right to pursue knowledge and understanding—in the same legal weight as the rights to freedom of opinion and expression, a fair trial, and even the right to the highest attainable standard of health.

From an international human rights law perspective, State obligations include three domains as articulated by the International Bill of Human Rights—to respect, to protect, and to fulfill.^32^ The latter has been codified in the ICESCR^33^ including setting legislative reparatory measures should these rights be violated. And the lack thereof would be a breach of a State’s obligation even if the State was not the perpetrator of the right violation. The States’ obligation extends to cover private entities within their region in the event of a violation of the RtS.^34^

Recognizing the RtS as a human right imparts a distinctive standing for scientists in society. More than ever, scientists are in the spotlight, which affords them the authority and responsibility to better humanity as a common good by augmenting their roles in policy-making that channel the implementation of benefits of their scientific progress that serves the public interest. Together with Article 2 of ICESCR, it adds a further assumed obligation on developed states to provide scientific and technical assistance and facilitate access across borders to essential information and technologies for the ‘full realization of the rights recognized in the present Covenant’.^35^

Other international rules exist but are deemed soft law instruments such as the 1975 UN Declaration on the Use of Scientific and Technological Progress in the Interests of Peace and for the Benefit of Mankind.^36^ It seeks to improve the conditions of peoples and nations and refrain from the potential for violation of human rights through misuse of science causing social problems or threaten human rights and fundamental freedoms. It also included non-discrimination and international cooperation that engender the use of the results of science and technology for the interest of peace and security and for economic and social development. Added to that are the 1997 Universal Declaration on the Human Genome and Human Rights,^37^ the Universal Declaration on Bioethics and Human Rights,^38^ 2005 Universal Declaration on Bioethics and Human Rights,^39^ the 2017 Recommendation on the Status of Science and Scientific Researchers,^40^ and 2021 Recommendation on Open Science.^41^ The Convention on Human Rights and Biomedicine of the Council of Europe, which entered into force in 1999, is the first international legally binding instrument designed to protect human rights in the field of biomedicine with its additional protocol. It includes provisions to protect this right in its preamble. Its protocol concerning biomedical research, which entered into force in 2007, goes further to ‘…guarantee everyone, without discrimination, respect for their integrity and other fundamental rights and freedoms with regard to any research in the field of biomedicine involving interventions on human beings’.^42^ But despite the plethora of regional and international normative tools that furnish expectant duties from its signatory governments,^43^ the UN has not established indicators or developed standards determining their respective obligations to realize this right, partly due to vagueness and lack of clarity/specifications in exactly what liberties and responsibilities derive from this right. Another is that the body of economic, cultural, and social aspects of today’s science enterprise has significantly evolved since first conceived such that contemporary international legal frameworks no longer adequately encompass it and, consequently, lack evidenced-based policy-making. To address this, concerted efforts were made that included UNESCO’s 2009 Venice Statement on the Right to Enjoy the Benefits of Scientific Progress aimed at ‘clarifying the normative content of the right to enjoy the benefits of scientific progress’.^44^ It followed the 2012 UN Human Rights Council special rapporteur in the field of cultural rights report, which shed light on the normative content of the right and stressed on the need for ‘a participatory process be adopted to enhance further the conceptual clarity of the RtS and related obligations’.^45^ In 2020, the CESCR published a General Comment No 25 on Article 15 on the right to enjoy the benefits of scientific progress and its applications as a further step to clarify the interpretation of this right thus reviving its normative stature.^46^ But as it is legally non-binding, ‘its de facto legal force and impact depends on how convincingly and persuasively it is argued’.^47^ Along those sentiments, some argued that it did not offer a comprehensive interpretation of this right.^48^ Nonetheless, it is widely regarded as the first to consider the RtS in depth that added to its normative legitimacy.

At an EU level, human rights are enshrined in the Charter of Fundamental Rights of the EU, which is a primary EU law. Although the RtS is not explicitly mentioned in the Charter, it includes the right not to be discriminated against in participation in cultural life. Article 13 (Freedom of the arts and sciences) of the Charter indirectly addresses this right: ‘The arts and scientific research shall be free of constraint. Academic freedom shall be respected’.^49^ But this comes with limitations that pose restrictions on its applicability predominantly relating to other Articles within the Charter. In the scientific terrain, these include the right to human dignity (Article 1), the right to the integrity of the person (Article 3), protection of personal data (Article 8), and the right to property in relation to intellectual property (Article 17). It is also worth noting that the scope of its application lies in Member States when implementing EU law as outlined in Article 51 of the Charter and not solely domestic issues.^50^ Article 168 Treaty on the Functioning of the EU (TFEU) affords the EU the supranational coordination and harmonization of Member States by supporting and supplementing its actions for the protection and improvement of human health and the protection of human rights.^51^ Member States are obligated to monitor the protection of this right, which is overseen by the European Network of National Human Rights Institutions. Specific to the conduct of clinical research are Regulation No 536/2014 of the European Parliament and of the Council of 16 Apr. 2014 on Clinical Trials on Medicinal Products for Human Use,^52^ and European Commission, European Charter for Researchers.^53^ Unlike EU regulatory framework, violations of any aspect of the RtS are subject to legally binding adjudications by the European Court of Human Rights (ECtHR),^54^ the international court of the Council of Europe and of which the EU member states are signatory to, whether at the individual (including private entities) or State level. Therefore, it follows that the contribution of the RtS is arguably the consequential normative tool to navigate the pervasive inequalities that hinder access to innovative cancer therapies.

Despite the aforementioned legal tools, it can be argued that the justiciability of this human rights category (economic, social, and cultural rights), of which RtS belongs to, lies in being a positive right where states have a duty to act to preserve that right under the ECtHR.^55^ The issue of direct enforceability of such right remains a highly contested issue. For one, it can be argued toward fully supporting a decision to pronounce state responsibility for its violations of Article 15 based on the interrelation and indivisibility of human rights with RtS being an implicit right much like the right to health. Alternatively, one can argue that its legal ambiguity deters direct justiciability for possible violations, which they might be found responsible by the ECtHR. The collective normative consequences of the international and EU laws resonate the former argument with progressive realization of three key issues afforded protection by Article 15 of the ICESCR—science, enjoyment of its benefits, and the participation for the fulfillment of international human rights obligations. However, these fall short of implementation with there still being a significant gap in the evolving interpretation of current normative frameworks that decide accountability and violations of ‘implicit’ human rights under international and EU laws but also their direct justiciability for the protection of science as a human right.

## III. THE HUMAN RIGHT CASE FOR CANCER RESEARCH: WHY IS IT RELEVANT IN THE EU?

Drug discoveries and novel technologies that shaped the latest practice-changing trends in innovative approaches to cancer treatment stem from basic, translational, and clinical research that attempts to understand the fundamental basis and mechanisms underlying cancer.^56^ These have been pivotal in the breakthroughs seen in groundbreaking cancer therapy that led to dramatic changes in cancer outcomes, including the progressive decline in overall cancer incidence and mortality rates^57^ and a significant improvement in the number of individuals who are living longer and fuller lives following a cancer diagnosis. It also set the foundation for precision oncology.^58^ Collectively, these underscore the value of science in cancer care. But why is this particularly relevant for the EU? The European Cancer Information System reported 2.7 million people newly diagnosed cancer cases each year with 1.3 million dying from it in Europe alone. Without intervention, the former is predicted to rise to 5.33 million by 2040.^59^ Hence, cancer became a significant public health concern. Cancer research is one the fastest developing and consequently the heaviest funded domains of science.^60^ So, it is no surprise that it gained priority status within the pharmaceutical industry that disproportionately skewed its research approach toward this field as seen with top pharmaceutical companies heavily investing their market on oncology.^61^ Affording the successes of such shift in the scientific industry comes with a price, literally.^62^ In 2024, the projected global oncology drugs market size was valued at $220.80 billion and expected to rise to $518.25 billion by 2032, with the EU only second to North America in its share in the global market.^63^ Pharmaceutical companies hike drug prices to leverage their budgetary distress of research and development costs, a move that draws wide criticism.^64^ High costs of new cancer drugs are one of the major barriers that limit affordability and consequently access to innovative treatments and technologies—a human rights issue in its own right protected under a ‘good faith’ interpretation of ICESCR (Articles 12 and 15(2)). This feeds into the problematic discrepancies seen in the outcomes of patients with cancer worldwide that further deepen the inequalities and perpetuate discrimination and marginalization not limited to LMIC but also EU Member States.

But undertaking and engaging in cancer research *per se* does not necessarily help curb cancer care disparities among and within countries. It is widely acknowledged that innovative cancer therapies are not reaching those who most need them in time or sometimes even ever at all.^65^ The average time required to develop a typical innovative drug is 9 years.^66^ This includes the time required to develop and implement first-in-human clinical trials, mandatory regulatory approval by the relevant authorization agencies, and health technology assessments. Consequently, access to *vis-à-vis* availability and affordability of much-needed cancer treatment is delayed. While the potential real-world impact of such delays has never been quantified, it can be extrapolated from potential prospective efficiencies. A recent analysis has shown that shortening the length of time between a positive Committee for Medicinal Products for Human Use recommendations and EC decision to grant marketing authorization by 12 days would result in an estimated 3300 years of potential lives (YPL) saved and 4200 YPL if this was increased to 15 days.^67^ Additionally, most pharmaceutical companies tend to swerve their research agendas toward the more lucrative novel targeted drugs and therapies, such as immunotherapy and gene therapies. Not only are these globally inaccessible, but they are also unlikely to be affordable for patients, particularly in LMIC. This fundamental ethical principle to enhance cancer treatments is for reputational and financial gains for the less scrupulous agents who claim to protect the public’s health.

Equity in the access of novel agents is a requisite for realizing health equity that lends itself to best cancer management. But the construct of the cancer research biosphere driven by the current drug market, which is predominantly privately funded, makes simpler research approaches, such as approved drugs repurposing,^68^ rather void given the lack of monetary incentives, thus prioritizing financial profits over health benefits. This paves the way to the egregious anticompetitive practices that feed into the predatory pricing of much needed cancer drugs.^69^ It can also lead to the monopolization of certain anticancer drugs as seen in some cases of advanced incurable cancers with decidedly limited treatment options.^70^ Added to that, their research and innovation (R&I) spillover is less impactful than publicly funded R&I when it comes to national aggregate productivity growth and that by 3-fold.^71^ Furthermore, in clinical trial settings, much of the focus of trial designs for novel interventions is centered on outcomes based on the statistical significance of pre-defined endpoints as it guarantees drug approval (at times even accelerates it) and consequently financial return. But this approach does not necessarily lead to clinically relevant outcomes. A study published by researchers from the Netherlands revealed that between 1995 and 2020, 41 per cent of oncology drugs approved by the European Medicines Agency were ‘negative or non-quantifiable’; ie of limited or no added benefit.^72^ A similar finding was noted in its US counterpart, the Food and Drug Administration.^73^ Existing international human rights law and international biomedical law instruments have failed to address these issues and stem the consequential tide of inequitable access to cancer drugs. Extending the RtS to tackle such issues could benefit society by allowing them to reach those most in need. Under this approach, disadvantaged and marginalized individuals particularly those living in developing countries would have a right and their respective states would allow the availability of these therapies. Researchers must perform scientific activities in the public interest. It is indisputable that the inequalities to access to cancer research benefits not only persist but are also deeply entrenched.

One of the main challenges for EU governments and policy-makers that stalls their efforts to ensure timely access to innovative cancer treatments and technologies is contending with rising demands on already cash-strapped public healthcare services and the often sparse evidence to support their safety and efficacy as noted above. Inequitable distribution of research efforts and resources directed toward the disadvantaged and marginalized communities has long been considered another major issue in global health research.^74^ This is not peculiar to cancer research or the EU. The EU has prioritized cancer research through its Cancer Mission initiative together with the Beating Cancer Plan, which launched in 2021 and reinforced at the Conference on the Future of Europe in 2022 with an emphasis on cancer health and wellbeing as part of a pan-European effort through integrated research beyond the political boundaries of the EU27 among its other objectives. Despite these efforts, there remains significant gaps for Europe, both for the Beating Cancer Plan and the Cancer Mission, in the inequalities that persist within the cancer health domain in much of central and eastern European countries. And as the EU took interest in investing in cancer R&I by around €3 billion since 2007^75^ in its efforts to curb the disease’s rising health and its associated economic burdens, so has the privately funded big pharma, which overtook the publicly funded research domains. A recent investigative report showed that in 2022, 85.9 per cent of the European Medicines Agency’s (EMA), Europe’s medicine regulator, operating revenue came from big pharma money, raising several concerns of unchecked conflict of interest from the industry.^76^ But also, it reflects on how big pharma has become a key player in driving the R&I pace and agenda^77^ that tends to steer away from those focusing on cancer control and prevention and shifting it toward drug development thus monopolizing and inflating the pricing power. It is hoped that the proposed reform of the core EU General Pharmaceuticals Legislation^78^ (Directive 2001/83/EC^79^ and Regulation 726/2004^80^) with its so-called ‘3 As’—Affordability, Accessibility, Availability goals and the EMA’s revised rules on handling competing interests following the recent court ruling^81^^,^^82^ would address the wider systemic issues that feed into social injustice.

The iterated concerns of the RtS are not exhaustive. Its implications are not limited to individuals benefiting from its progress. Rather, it extends to future generations and their societal issues that may raise concerns for violation of the future rights, *qua* intergenerational justice, to live as autonomous individuals through access to such benefits.^83^ Such a violation creates a human rights burden as involved individuals do not truly recognize the risks and benefits, including long-term ones, of establishing equity and justice. Granted, the legal standing of extending the RtS to next generations is a highly contentious preposition but not at all far-fetched and brings us to questions of responsible science, its diplomacy, and policy-making. Thus, engaging human right to science imperatives and the very text of right of ‘everyone to benefit from scientific progress’ might be argued as explicitly recognizing considerations for the rights of future generations. Further concerns relate to shifting sociological norms with the resultant ramifications of inequality at the individual and country level as such technology is readily accessible to all. Accordingly, the RtS can be viewed as an instrumental tool to realizing the right to health, among others.

## IV. THE RTS AND THE PERVASIVE PROBLEM OF ACCESS TO CANCER INNOVATION IN LMIC

LMIC continue to struggle with cancer health disparities primarily due to barriers in access to scientific innovation and its associated high costs. Though burgeoning globally, LMIC are particularly inflicted as they grapple with double the burden. For one, it has one of the highest overall cancer death rates with 70 per cent of the 10 million estimated deaths reported to be due to cancer in 2020 alone^84^ even though the overall age-standardized cancer incidence rate is lower in LMIC.^85^ And it is estimated that by 2030, around three-quarters of all cancer deaths will be in LMIC.^86^ Added to that, the unequal global order, exacerbated by EU’s trade policy, hinders LMIC’s ability to access these innovative treatments and further impeded by the limited opportunities for access afforded under global patent law.^87^ There is an increasing reliance on high-income countries (HIC) for answers to the regional cancer problems and a lack of alternative approaches to problem-solving that take local contexts into account feeds into the discordance outcomes. Besides, funding and undertaking cancer research within HIC will not necessarily generate the relevant country-specific information needed to help bridge/leverage the disparity gap within LMIC.^88^ Furthermore, current paradigms of scientific advancement do not provide sufficiently robust models to challenge the status quo required to address such disparities.

Many in LMIC do not benefit from scientific and technological advancements, as only a fraction of the knowledge and technology is available, accessible, and/or affordable in these countries. Among other legal instruments outlined above, Article 13 of Charter of Economic Rights and Duties of States^89^ includes a right of a State ‘to benefit from the advances and development in science and technology for the acceleration of its economic and social development’ in addition to the promotion, cooperation, and transfer of technology to developing countries noting such obligation lies within States rather than individuals. Granted, it establishes the onus on governments of HIC to instigate the necessary provisions that affords LMIC to benefit from the scientific advances of the former States. While viewed as a means by which LMIC can overcome the discordance in access to innovation, the Charter of Economic Rights and Duties of States opted for ‘fulfillment in good faith of international obligations’ as its governing principle,^90^ which renders it a contractual obligation rather than international law.^91^ In the absence of apparent reciprocity of palpable interests, HIC would see no reason to be legally bound to the obligations set out by the Charter. One incentive though comes from the pervasive financial burden exacerbated by an aging global population and the insatiable social appetite for better outcomes that cancer care causes which even HIC are not immune to. Research into means by which this burden can be curbed without adversely affecting outcomes could address this and LMIC could set a precedent to these types of research. Hence, international solidarity and collaboration are central in this regard if scientific advances against cancer benefit all patients, regardless of their socioeconomic status or geographic location.

## V. THE HISTORICAL DIVIDE BETWEEN THE RTS AND INTELLECTUAL PROPERTY RIGHTS

Article 15 of the ICESCR offers a theory of RtS in preserving the knowledge of commons by drawing on accepted methodologies of human rights interpretation and the views of legal and economic scholars. It then translates this into guidance for policy-makers implementing human rights obligations and supporting jurists when adjudicating right-based challenges to intellectual property rights (IPR) laws. But regardless of its legal status, the RtS seems to have had negligible tangible impact in averting or reparatory effect on disparities in cancer care caused by a range of domestic and international factors, among which legal and philosophical constraints that impede the further expansion of protectionism in IPR law is a major one. RtS and IPR operate as two relatively distinct domains—property in information and scientists’ efforts to generate, develop, disseminate, and share that information. But with public interest in science steadily growing, the inevitable tensions highlighted the question of justice in the face of the question of individual autonomy—whether it is morally right that an individual’s autonomy is curtailed for the sake of societal justice. Some degree of incongruity regarding how to balance competing concerns is inevitable given the inherent tensions within the right, noting that aspirations that science delivers benefits for humanity may equally risk science’s autonomy. Susan Sell described these tensions as ‘one between romantic notions of authorship and invention on the one hand, and utilitarian conceptions of incentives for creation and diffusion on the other’.^92^ This stems from the UDHR, ICESCR, and CFREU unintended *faux pas* of applying the same norms to two domains with competing interests—IPR and cultural rights. For instance, biopharmaceutical industries commonly use patents to protect their innovations’ intellectual property. While this may serve artistic and entertainment industries’ IPR interest, as it keeps the dissemination of their respective work in check, the contrary is true in the interests of science, where sharing the work as early and widely as possible is the goal. One such instance was adapted in the UK on issuing Control of patient information (COPI) Notices that afforded the legal grounds for sharing confidential patient information with proscribed organizations for COVID-19 purposes. Although it was time-limited, it set a precedent for the lawful justification on the normative grounds of the RtS on prioritizing public interest over confidentiality laws in the event of health threat to the general population.^93^ On a wider scale, the Sub-Commission on the Promotion and Protection of Human Rights in 2000 adopted a resolution calling for the prioritization of human rights over trade (resolution 2000/7).^94^ This afforded a rebalance of international IP governance and opened the ‘access to knowledge’ movement to public interest groups and developing countries. Subsequently, the Geneva Declaration on the Future of the World Intellectual Property Organization (WIPO) called for alternative policy approaches to promote innovation and creativity without the social costs of privatization.^95^ At an EU level, Molnár-Gábor argued that revisiting the interpretation of secondary EU data protection law through a RtS lens can be utilized as a blueprint for establishing a governance framework for health-related data processing of biomedical knowledge by relevant actors in the field of science to promote data-sharing in the context of this right.^96^

The role of ethics, human rights, and human dignity in IPR has been subject to many debates and serious concerns over the past decade and are rarely a direct provision in international law. This is likely related to the utilitarian nature of IPR laws that potentially undermine leeway for access to patent-protected medicines and its varied indeterminate approach to its regulation worldwide. In the EU and despite the overarching EU law that may be interpreted by the European Court of Justice, legislation of IPR is subject to national rules within the terrain of individual Member States with laws principally based on enacting protection laws for the protection of data and facts.^97^ Hierarchical systems regulate IPR in the form of the 1994 Trade-Related Aspects of IPR (TRIPS) Agreement, WIPO and WIPO Copyright Treaty (WCT), which have effectively nullified some of the limitations and exceptions to the exclusive rights recognized in national IP laws.^98^ Despite this, concerns remain around whether such laws can further alleviate impediments to scientific research conduct and dissemination. With advances in digital technologies comes the added challenge of IP, which has led to a global response inclusive of the USA and the EU to legislative regimes akin to those of the WCT for the prevention of use of most limitations and exceptions and prevention of third parties from accessing scientific facts and data.^99^ This, unfortunately, further confounds access to basic knowledge and increasingly infringes the progress in scientific research.

Article 2 of the Oviedo Convention on Human Rights and Biomedicine states ‘The interests and welfare of the human being shall prevail over the sole interest of the society or science is to be fully respected. The interests and welfare of the individual should have priority over the sole interest of science or society’. Patent systems are generally envisioned to benefit society at large but often fall short when considering the downside of individual deprivation, which highlights its basic philosophy: utilitarianism. Therefore, it is ultimately the balance of rights to human dignity, privacy, and property and the potential for burden on research and society that needs to be considered in patenting biotechnology.

This paper does not engage with the broader IPR–RtS tensions as it requires more in-depth analysis beyond its scope and is delved into more comprehensively elsewhere.^100^ Instead, it offers a global legal and ethical discourse to balance potentially conflicting interests when addressing the question of equity.

## VI. THE INTERFACE OF HUMAN RIGHTS, SCIENCE, SOCIETY, AND POLITICS—REPARATIONS OF A NEGLECTED RIGHT

The role of science in society and the human rights–based approaches are increasingly being debated in polarized, politicized, and partial terms. Inequities across the cancer research continuum stem from historical systemic socio-political injustices and lead to unfavorable differences in social determinants of health in underserved populations that perpetuate the same institutional and societal injustices that impede progress in oncology outcomes. This perhaps lends itself to the fact that the RtS remains a neglected and lesser-understood human right at national and international levels. Persistent global inequalities perpetuated by the trending privatization of science and current IPR laws hinder just and equitable responses to access science and its benefits.

Beyond defending IPR of scientific work, upholding the essence of RtS is arguably key to resolving the fundamental challenges to equitable and just access to the benefits of cancer research. Much like how other human rights are conceptualized, there needs to be a multi-faceted approach that encompasses a panoptic, systematic contextualization of the RtS that offsets socio-political schism tendencies for commercial interests. Science, politics, law, and society have been ubiquitous features of human rights with the current understanding of modern legal rights being shaped and delivered through state laws. Rights increasingly require the affirmative mobilization of resources that are notably difficult to provide simultaneously. Rights also require otherwise costly or scarce resources, such as access to high-cost medicines and other associated healthcare services. But as Moyn poignantly argues, the case for human rights in addressing economic fairness is ‘unambitious in theory and ineffectual in practice in the face of market fundamentalism’s success’, ‘contributed little of note, merely nipping at the heels of the neoliberal giant’, and ‘even perfectly realized human rights are compatible with radical inequality’, which eloquently summed up the title to his seminal book ‘Not Enough: Human Rights in an Unequal World’.^101^ He drew attention to the distinction between sufficiency, ‘how far an individual is from having nothing and how well she is doing in relation to some minimum of provision of the good things in life’, and material equality, ‘concerns how far individuals are from one another in the portion of those good things they get’, which he also refers to as ‘distributive equality’, ‘economic justice’, or just ‘equality’. This stands true even in the scientific terrain despite proliferative efforts to address them. So, whose duty is it to make lifesaving cancer therapies accessible and affordable particularly with the ever-upward spiraling costs of research?

States, under the RtS (Article 15(1)(b) of the ICESCR), have the following obligations: to mobilize public funds in the interest of serving the benefits of scientific progress where public health needs are required and mitigate usuriously high medicinal prices and international collaboration. The CESCR, in its General Comment No 25 on equality, indicates specific State obligations related to science, which include taking ‘steps, to the maximum of their available resources, for the full realization of the right to participate in and to enjoy the benefits of scientific progress and its application’ and ‘overcome persistent inequalities in scientific education, design, and implementation policies’. And so, how can this be moved forward? Despite the increasing literature that brings the RtS to fort, governments continue to either disregard establishing robust policies that regulate its implementation^102^^,^^103^ and/or abuse existing fiscal policies that exacerbate economic inequality for political gains,^104^^,^^105^ and COVID-19 pandemic was one example.^106^ Harnessing science, policy, and law to deliver actionable policies that respect the RtS and its benefits as articulated by the ICESCR allows for the addressing of the ‘wicked problems’ that come with implementing distributive justice.^107^ Advocating science and its progress alone are insufficient to deliver the goals set by UDHR, ICESCR, and CFREU; the law must play a central role in shaping and implementing actionable policies. Accountability and transparency are paramount particularly among the biopharmaceutical industry. A seminal study showed that R&I costing of oncology drug development using precision approach by biopharmaceutical companies were $1 billion cheaper than non-precision drugs—a finding that could only be corroborated in companies that had ‘rigorous and transparent accounting standards’.^108^ Yet, despite several international laws, treaties, and national regulations that seek to address equity and accessibility to science, many states still do not observe the basic principles of this right. Advocating a human rights–based approach rather than a risk-based one that ‘draws on a “common fund of knowledges” to formulate anticipatory responses’ should be adopted if an effective response to the regulatory challenges posed is to be realized.^109^

There are three key issues linked to science—society, law, and policy. Each show why RtS governance is a complex, interdependent, and dynamic regulatory space, which needs multidisciplinary attention. There are, however, caveats that need to be contended with. Although the problem of inequity is global, the EU stands central in addressing the problem not just by being a conglomerate of developed countries but also, given its track record in R&I, its claimed renowned global stature as the human rights gatekeeper and a key player in global health governance.^110^ Accordingly, EU legal jurisdictions and ethical frameworks as the primary references for the legitimacy of the RtS are the focus of my recommendations. The influence of the rest of the world’s legal norms and/or lack thereof can impact the implementation of remedial measures given the wider legal differences in many jurisdictions around norms, such as the interpretation of specific human rights and the legitimacy of international laws.

First, priorities should be set aimed at harnessing maximum benefits to patients over profit gains. The interpretation of existing human rights can be geared toward more aggressive policy-making decisions on the redistribution of scientific benefits. Depending on the relevant legal framework, setting priorities requires harnessing research-driven advances based on robust scientific evidence and tangible scientific feasibility that is economically and socio-politically acceptable but also rationed toward the real need for novel anticancer agents in line with Article 114(3) TFEU.^111^ However, interpretations need to be tempered with attention to specific contexts and opportunities as otherwise, it can entrench health injustices, undermine the just access to innovative therapies, and perpetuate inequity.

A way to address this is to recognize that science, law, and policy need to be brought closer for the so-called ‘apartheid’ in access to innovation to be redressed. It must adopt a solution-oriented approach with equity-focused implementation science that sets pragmatic end-points for real-world applicability and not economic dividends. This includes explicit emphasis on the unmet needs of the underserved and marginalized patients that drive inequity. For instance, where a reasonable standard of care already exists, alternative strategies that focus on optimization/refinement of existing interventions such as repurposed drugs or alternative dosing strategies will likely offer a better impact on patient care than developing an expensive drug. As an example, a health economic analysis reported that treatment breaks of one of the ‘conventionally continuous’ targeted therapies in a subtype of metastatic colorectal cancer saved the UK National Health Service around £1.2 billion over the study cohort’s lifetime while potentially improving tumor responsiveness and reducing drug toxicity.^112^

Another strategy would be to invest in research of generic and biosimilar drugs that are cheaper and therefore improve access.^113^ Both strategies may lower costs without adversely impacting outcomes but require leveraging the frequent opposition from the biopharmaceutical industry. To navigate the challenges of the latter, reputable scientific organizations such as the European Society for Medical Oncology, European Organization for Research and Treatment of Cancer, and the European Association for Cancer Research, which have a long and prominent history of supporting impactful cancer research, must double down their efforts in reaching out to EU lawmakers and health policy-makers about the vital role pharmaceuticals and clinical research regulatory norms have in advancing responsible science. The EU’s overarching governance architecture is also crucial in reforming the market-optimized Europe with emphasis on innovations with greater effectiveness, equity, and justice that serve the public’s unmet needs over one that prioritizes the ‘pharmaceuticalization’ of science. It needs to adapt its incentive models to reward institutions for their compliance in research that is inclusive, equitable, and affordable. At an individual level, clinician scientists need to work on diversifying funding sources from non-profit organizations that are free from the conflict of interest of commercial bias with the support of their academic institutional research infrastructure for ethical and human rights–friendly research.

Second, bolstering a multi-faceted approach to coordination and accountability that ensures alignment with the human values of the RtS. Inequity in science arises from nearly all stakeholders involved. Hence, public and academic institutions, private sectors, and civil society organizations are implicated and consequently need to contribute to developing scientific solutions. Government structures need to establish provisions that harness cross-economy goals through coordinated policy-making that encompasses the varied roles of science, law, policy, and accountability to deliver on all ICESCR right-to-science objectives. This should be reinforced by catalyzing resources and emboldening scientific advisory and regulatory bodies through effective dialogue on policy-making that prioritizes public interest and that aligns with the human rights obligations articulated in Article 15 of the ICESCR while mandating accountability, participation, and transparency in decision-making through all government levels regionally and internationally. It must be recognized as an interrelated, multilayered socio-economic policy issue at the national and inter-State levels through dialogue rather than an isolated, localized human right problem or mere scientific data collection. Notably, the aspirations of one country’s scientific advances should not be undermined by choices made by another. Robust governance across countries should include obligations for knowledge sharing and mutual working to reduce inequity effectively. This can be achieved through unified standards, adherence to legal provisions, and an enabling environment for participation in science and the sharing and dissemination of critical knowledge to relevant stakeholders that ultimately serve the public interest. Scientists too should play a central role in the delivery of these objectives for effective policies.

Third, health economics and technology assessments centered on human rights principles. Normative and ethical debates should include a Human Rights Impact Assessment to evaluate the potential sequel of innovative cancer therapy applications and the quality of the scientific data likely to be generated may have on human rights. This should not just be limited to those who benefit directly from it but also extend to those not participating in research, ergo generalizability, for when the research drug is licensed. One such strategy is the governments’ advocating for an approach that adopts anticipation entitlements and duties—one that weighs up ‘the beneficial and harmful impacts of scientific and technological progress’ envisaged by the ethos of the human RtS to achieve ‘beneficial, responsible, and participatory anticipation’ for enriching this right.^114^ Considering the recognized profound under-representation of underserved and marginalized cancer patients in cancer clinical trial research, this step would better inform trial design and setup that addresses the genuine unmet medical and health needs and consequently advance equity and that at far less capital than when conducting research in HIC. Channeling these ethical standards, and through them human rights principles consistent with the EU’s ethos that it prides itself for, needs to be at the center of its normative regulatory regime and code reform.

Fourth, a call for legal and policy scholars to adopt a progressive critique of expertise approach for advancing the legal standing of RtS in international human rights law. McHenry articulately surmises that ‘industry is not programmed to do critical, scientific testing; rather it is designed to circumvent the process, to minimize financial loss, eliminate competition, and suppress criticism’.^115^ Science cannot progress if there are restrictions or barriers to test all available knowledge beyond only delectable thinking as this would paralyze any workable progress in the scientific ecosystem. Needless to mention, it is inconsistent with responsible science. Progressive critique of expertise allows for the collaboration of law and science in reaching a new evidence-based, expert-led factual consensus that affords scope for providing solutions to health injustices. It serves in achieving justice as it ‘…depoliticizes decision making in the healthcare space and keeps them in the realm of the technocratic’,^116^ which lends to its objective in seeking equitable access to science through *active* evidence-based scientific deliberation and expertise and not mere deference to it. Consequently, it helps inform policy-makers in reaching better solutions toward just distributional outcomes in lawmaking on science and R&I resources.

Notably, these recommendations presented are mainly voluntarily with self-regulating governance; herein presents the difficulties that are ever present in the lack of meaningful definition of the RtS. Nonetheless, they are intended to serve as a reference for stakeholders in cancer research and law to explore the potential implications of innovation to cancer management and control and to promote the vision of a ‘just society’ that nurtures ‘science for all’. Fundamentally a legislated line is required and universally accepted as in the public interest and serves as a public good.

## VII. CONCLUSION

Neglecting the RtS is an ever-present concern in achieving equity within cancer research. And now more than ever, this has been lamented by the increasingly expensive and unsustainable clinical research enterprise notwithstanding the impending consequences of research funding cuts^117^^,^^118^ and/or geopolitical uncertainties and interferences.^119^  ^120^ There are multiple challenges to its realization among which is the lack of socio-political will to set robust cancer health policies. Pragmatic policies, guided by human rights principles and international law order that are aimed at promoting universal access to science and its benefits and consequently feed into serving the public interest, should be adopted to help shape and prioritize a response to meeting legal obligations and steer away from policies driven by commercial interests. Those legal obligations cannot be static in delivering equity outcomes given the increasingly dynamic nature of cancer research, with a growing, intersecting influence on society, scientists, and the judiciary in molding policy trends. It should not just be a matter of increasing legal standards. Henceforth, a participatory collaboration and partnership across the globe at citizen, public, and private sector levels is paramount for a more transformative focus on advancing scientific progress. Governments and states must work toward creating a dynamic regulatory framework at the science–society–law–policy interface conducive to the principles of human rights with transparency and accountability at its core. It should encourage and reward innovative approaches toward accelerating global science delivery. After all, investing in cancer R&I is akin to investing in a healthier and more productive society. In the words of the UN High Commissioner for human rights Volker Türk, ‘Human rights are not simply laws or an ideology—they are the key to survival of our species. By taking them seriously—as intended by the Universal Declaration 75 years ago—we can transform our current trajectory to safeguard the well-being of our planet for us and future generations.’^121^

## Footnotes

1 Elmore LW, et al., *Blueprint for Cancer Research: Critical Gaps and Opportunities*, CA Cancer J Clin. (2021). https://doi.org/10.3322/caac.21652

2 Zhang, Y., Zhang, Z., *The History and Advances in Cancer Immunotherapy: Understanding the Characteristics of Tumor-Infiltrating Immune Cells and their Therapeutic Implications*, 17 Cell Mol Immunol. 807–821 (2020). https://doi.org/10.1038/s41423-020-0488-6

3 Fountzilas, E., et al. *Convergence of Evolving Artificial Intelligence and Machine Learning Techniques in Precision Oncology*, 8 Npj Digit. Med. 75 (2025). https://doi.org/10.1038/s41746-025-01471-y

4 Lu, C.Y., Terry, V. & Thomas, D.M., *Precision Medicine: Affording the Successes of Science*, 7 Npj Precis. Onc. 3 (2023). https://doi.org/10.1038/s41698-022-00343-y

5 Natasha B. Leighl et al., *An Arm and a Leg: The Rising Cost of Cancer Drugs and Impact on Access*, 41 Am Soc Clin Oncol Educ Book e1–e12 (2021). http://dx.doi.org/10.1200/EDBK_100028

6
National Academies of Sciences, Engineering, and Medicine; Health and Medicine Division; Board on Population Health and Public Health Practice; Committee on Community-Based Solutions to Promote Health Equity in the United States; Baciu A, Negussie Y, Geller A, et al., editors. Communities in Action: Pathways to Health Equity. Washington (DC): National Academies Press (US); 2017 Jan 11. 3, The Root Causes of Health Inequity. Available from: https://www.ncbi.nlm.nih.gov/books/NBK425845/

7 CESCR, General Comment No. 25, UN Doc. E/C.12/GC/25 (2000).

8 Tenni, B. et al., *What is the Impact of Intellectual Property Rules on Access to Medicines? A Systematic Review*, 18 Globalization and Health 40 (2022). https://doi.org/10.1186/s12992-022-00826-4

9 Flear ML, *The EU Clinical Trials Regulation: Key Priorities, Purposes and Aims and the Implications for Public Health*, 42 J Med Ethics 192–82,016. http://dx.doi.org/10.1136/medethics-2015-103,258.

10 Case C-66/18, European Commission v Hungary, E CLI:EU:C:2020:792.

11 CJEU, 18 Oct 2011, C-34/10 (Brüstle), ECLI:EU:C:2011:669.

12 Universal Declaration of Human Rights, G. A. Res. 217A (III) (1948), art.27.

13 International Covenant on Economic, Social and Cultural Rights, G.A. Res. 2200A (XXI) (1966), art.15.

14 European Commission: Directorate-General for Health and Food Safety | Directorate-General for International Partnerships. EU Global Health Strategy — Better Health for All in a Changing World, 30 November 2022; available at https://health.ec.europa.eu/publications/eu-global-health-strategy-better-health-all-changing-world_en

15
https://www.whitehouse.gov/presidential-actions/2025/01/withdrawing-the-united-states-from-the-worldhealth-organization/

16 J. Symonides, *Cultural Rights: A Neglected Category of Human Rights*, 50 International Social Science Journal 559 (1998).

17 E. McKiernan. ‘University research: if you believe in openness, stand up for it,’ Guardian (Aug. 22, 2014), https://www.theguardian.com/higher-education-network/blog/2014/aug/22/university-research-publish-open-access-journal#:~:text=Access%20to%20information%20is%20a,open%20data%20and%20open%20science

18 C. Romano and A. Boggio. “Preface, in The Human Right to Science: History, Development, and Normative Content” (Oxford Academic, 2024), p.17.

19 W. Schabas, *Looking Back: How the Founders Considered Science and Progress in their Relation to Human Rights*, 4 European Journal of Human Rights 504 (2015).

20 Mann, Sebastian Porsdam, Helle Porsdam, and Yvonne Donders, *“Sleeping Beauty”: The Right to Science as a Global Ethical Discourse*, 42 Human Rights Quarterly 332–356 (2020). https://dx.doi.org/10.1353/hrq.2020.0020

21 Weston, B. H. “Human rights.” Encyclopedia Britannica, Jan. 23, 2025. https://www.britannica.com/topic/human-rights

22 G. Irzik and F. Kurtulmus, *Distributive Epistemic Justice in Science*, 75 The British Journal for the Philosophy of Science 2 (2024).

23 L. King, *Concepts, Conceptions, and Principles of Justice*, 10 Political Science Faculty Publications (2012).

24 American Declaration of the Rights and Duties of Man, E/CN.4/122 (1948).

25
*Supra* note 13.

26 Saul, Kinley, and Mowbray, *Article 15: Cultural Rights*, in The International Covenant on Economic, Social and Cultural Rights: Commentary, Cases and Materials 1175 (2014).

27
Cesare P. R. Romano and Andrea Boggio. The Human Right to Science. History, Development, and Normative Content. Oxford University Press (2024).

28
*Supra* note 13.

29 European Social Charter, *A Step by Member States to take Further Commitments under the Charter*, (2024), https://rm.coe.int/en-vilnius-declaration/1680b0dcf3

30 van Beers BC, *Rewriting the Human Genome, Rewriting Human Rights Law? Human Rights, Human Dignity, and Human Germline Modification in the CRISPR Era*, 7 J Law Biosci. lsaa006 (2020). http://dx.doi.org/10.1093/jlb/lsaa006

31
*Supra* note 5.

32 International Bill of Human Rights, A/RES/217(III)A-E (1948).

33
*Supra* note 5, art 2.2.

34 Besson, S., *The ‘Human Right to Science’ qua right to participate in science*, 28 The International Journal of Human Rights 497–528 (2023). http://dx.doi.org/10.1080/13642987.2023.2251897

35
*Supra* note 5.

36 UNA (01)/R3, Res. 3384(XXX), Declaration on the Use of Scientific and Technological Progress in the Interests of Peace and for the Benefit of Mankind. Adopted at the 2400th plenary meeting, 10 Nov. 1975.

37 UN Educational, Scientific and Cultural Organisation (UNESCO), Universal Declaration on the Human Genome and Human Rights, -, 11 November 1997, https://www.refworld.org/legal/resolution/unesco/1997/en/28510

38 UNESCO Doc. 33 C/Resolution 15 (SHS/EST/BIO/06/1, SHS.2006/WS/14), 19 October 2005.

39 UNESCO (2005) UNESCO Universal Declaration on Bioethics and Human Rights. UNESCO, Paris.

40 UNESCO Doc. 39 C/Resolution 15 (UNESCO Recommendation), SHS/BIO/PI/2017/3 rev, 13 November 2017.

41 UNESCO (2021). *UNESCO Recommendation on Open Science* (p. 34). UNESCO. https://doi.org/10.5281/zenodo.5741832

42 Strasbourg 25/01/2005 - Treaty open for signature by the Signatories to Treaty ETS 164 (CETS No. 195).

43 Yotova, R., *Anticipatory Duties under the Human Right to Science and International Biomedical Law*, 28 The International Journal of Human Rights 397–415 (2023). http://dx.doi.org/10.1080/13642987.2023.2273289.

44 UNESCO, *Venice Statement on the Right to Enjoy the Benefits of Scientific Progress and Its Applications*, (2009). https://unesdoc.unesco.org/ark:/48223/pf0000185558

45 Human Rights Council, the Right to Enjoy the Benefits of Scientific Progress and its Applications: Report of the Special Rapporteur in the Field of Cultural Rights, UN Doc. A/HRC/20/26 (2012).

46
*Supra* note 2.

47 K. Mechlem, *Treaty Bodies and the Interpretation of Human Rights*, 42 Vanderbilt Law Review 905 (2021).

48 A. Mazibrada, M. Plozza, & S. Porsdam Mann, *Innovating in Uncharted Terrain: On Interpretation and Normative Legitimacy in the CESCR’s General Comment No. 25 on the Right to Science*, 28 The International Journal of Human Rights (2023).

49 Charter of Fundamental Rights of the European Union, C 326/02 (2007), art.13.

50 S. Peers et al., *Article 13*, in The EU Charter of Fundamental Rights: A Commentary (Bloomsbury Publishing, 2021) p. 405.

51 Consolidated version of the Treaty on the Functioning of the European Union - Part Three: Union Policies and Internal Actions - Title XIV: Public Health - Article 168.

52 Regulation (EU) No 536/2014 of the European Parliament and of the Council of 16 April 2014 on clinical trials on medicinal products for human use (2014).

53 European Charter for Researchers, the Code of Conduct for the Recruitment of Researchers, EUR 21620 (2005).

54
https://www.echr.coe.int/documents/d/echr/questions_answers_eng

55 European Convention on Human Rights No. 7 (2007).

56 N. Howlader et al., *The Effect of Advances in Lung-Cancer Treatment on Population Mortality*, 13 N Engl J Med 383 (2020).

57 Howlader N et al., *The Effect of Advances in Lung-Cancer Treatment on Population Mortality*, 383 N Engl J Med 640–6,492,020. http://dx.doi.org/10.1056/NEJMoa1916623.

58
*20 years of precision medicine in oncology*, 397 The Lancet 1781.

59 ECIS - European Cancer Information System. https://ecis.jrc.ec.europa.eu/explorer.php?$0-0$1-All$2-All$4-1,2$3-0$6-0,85$5-2022,2022$7-7$CEstByCountry$X0_8-3$X0_19-AE27$X0_20-No$CEstBySexByCountry$X1_8-3$X1_19-AE27$X1_-1-1$CEstByIndiByCountry$X2_8-3$X2_19-AE27$X2_20-No$CEstRelative$X3_8-3$X3_9-AE27$X3_19-AE27$CEstByCountryTable$X4_19-AE27, accessed on Nov. 15, 2024.

60
https://www.iqvia.com/insights/the-iqvia-institute/reports-and-publications/reports/global-oncology-trends-2024

61
https://www.statista.com/statistics/309711/oncology-market-share-of-top-ten-pharmaceutical-companies-worldwide/

62 Sarah Neville. *Pricy new cancer drugs offer patients hope but pile pressure on health budgets*. Financial Times. Sept. 8, 2024. Available on: https://www.ft.com/content/4dfc5540-da29-4701-b773-6e02b4c71a1e

63 Oncology Drugs Market Size, Share & Industry Analysis, By Drugs Class (Cytotoxic Drugs, Targeted Drugs, Hormonal Drugs, and Others), By Therapy (Chemotherapy, Targeted Therapy, and Immunotherapy), By Indication (Lung Cancer, Stomach Cancer, Colorectal Cancer, Breast Cancer, Prostate Cancer, and Others), By Dosage Form (Solid, Liquid, and Injectable), By Distribution Channel (Hospital Pharmacies, Retail Pharmacies, and Online Pharmacies), and Regional Forecast, 2024–2032. Last Updated: Oct. 28, 2024 | Format: PDF | Report ID: FBI103431. Source: https://www.fortunebusinessinsights.com/oncology-drugs-market-103431

64 Tay-Teo K, Ilbawi A, Hill SR, *Comparison of Sales Income and Research and Development Costs for FDA-Approved Cancer Drugs Sold by Originator Drug Companies*, 2 Jama Netw Open e186875 (2019). http://dx.doi.org/10.1001/jamanetworkopen.2018.6875.

65 Wilking, N. et al., *Achieving Equal and Timely Access to Innovative Anticancer Drugs in the European Union (EU): Summary of a Multidisciplinary CECOG-Driven Roundtable Discussion with a Focus on Eastern and South-Eastern EU countries*, 4 ESMO Open e000550 (2019). https://doi.org/10.1136/esmoopen-2019-000550

66 Brown, D. G. et al. *Clinical development times for innovative drugs*, 21 Nature Reviews Drug Discovery 793–794 (2021). https://doi.org/10.1038/d41573-021-00190-9

67 Vintura. *Every Day Counts. Improving Regulatory Timelines to Optimise Patient Access to Innovative Oncology Therapies in Europe*. November 2021. Available on: https://efpia.eu/media/636486/improving-regulatory-timelines-to-optimise-patient-access-to-innovative-oncology-therapies-in-europe.pdf

68 Schein CH, *Repurposing Approved Drugs for Cancer Therapy*, 137 Br Med Bull 13–27 (2021). http://dx.doi.org/10.1093/bmb/ldaa045.

69
https://www.statnews.com/pharmalot/2021/04/07/merck-austria-brain-cancer-antitrust/

70 Kira Le, *Crawling to the Cure: The Monopolization of the Breast Cancer Drug Market*, 25 MINN. J.L. Sci. & Tech. 185 (2023). Available at: https://scholarship.law.umn.edu/mjlst/vol25/iss1/7

71 Arnaud Dyevre. *Public R&D Spillovers and Productivity Growth*. Jan. 22, 2024 [Latest version]. Available on: https://www.ecb.europa.eu/press/conferences/ecbforum/shared/pdf/2024/EFCB_2024_Dyevre_paper.en.pdf

72 Brinkhuis F et al., *Added Benefit and Revenues of Oncology Drugs Approved by the European Medicines Agency between 1995 and 2020: Retrospective Cohort Study*, 384 Bmj e077391 (2024). http://dx.doi.org/10.1136/bmj-2023-077391

73 Liu ITT, Kesselheim AS, Cliff ERS. *Clinical Benefit and Regulatory Outcomes of Cancer Drugs Receiving Accelerated Approval*, 331 Jama 1471–1479 (2024). http://dx.doi.org/10.1001/jama.2024.2396

74 Yegros-Yegros, A. et al., *Exploring Why Global Health Needs Are Unmet by Research Efforts: The Potential Influences of Geography, Industry and Publication Incentives*, 18 Health Res Policy Sys 47 (2020). https://doi.org/10.1186/s12961-020-00560-6

75 European Commission: Directorate-General for Research and Innovation, *EU research and innovation in the fight against cancer*, Publications Office, 2021, https://data.europa.eu/doi/10.2777/072928

76 Leïla Miñano, Maria Maggiore, Manuel Rico, Catrien Spijkerman. *Big pharma influence hangs over Europe’s medicines regulator*. Investigate Europe. Published on 17 June 2024. Available on: https://www.investigate-europe.eu/posts/deadly-prices-big-pharma-influence-hangs-over-europes-medicines-regulator

77 Joseph M. Unger et al., *Patient Enrollment to Industry-Sponsored Versus Federally-Sponsored Cancer Clinical Trials*, 42 Jco 3917–3925 (2024). http://dx.doi.org/10.1200/JCO.24.00843

78 European Commission: Reform of the EU pharmaceutical legislation. 26 April 2023. Available on: https://health.ec.europa.eu/medicinal-products/pharmaceutical-strategy-europe/reform-eu-pharmaceutical-legislation_en#latest-updates

79 Directive 2001/83/EC of the European Parliament and of the Council of 6 November 2001 on the Community code relating to medicinal products for human use.

80 Regulation (EC) No 726/2004 of the European Parliament and of the Council of 31 March 2004 laying down Community procedures for the authorisation and supervision of medicinal products for human and veterinary use and establishing a European Medicines Agency (Text with EEA relevance).

81 Judgment - 22/06/2023 - Germany v Pharma Mar and Commission. Case C-6/21 P (Joined Cases C-6/21 P, C-16/21 P).

82 Judgment - 14/03/2024 - D & A Pharma v Commission and EMA Case C-291/22 P.

83 Meyer, Lukas, *Intergenerational Justice*, in The Stanford Encyclopedia of Philosophy (Summer 2021 Edition), Edward N. Zalta (ed.), available on https://plato.stanford.edu/archives/sum2021/entries/justice-intergenerational/

84 IARC, WHO. *Global Cancer Observatory* (2020).

85 Goss, P. E. et al., *Challenges to Effective Cancer Control in China, India, and Russia*, 15 The Lancet Oncology 489–538 (2014). https://doi.org/10.1016/S1470-2045(14)70029-4

86 IARC, WHO. Cancer Tomorrow. Global Cancer Observatory (2021). https://gco.iarc.fr/tomorrow/en

87 Perehudoff, K. et al., *Impact of the European Union on Access to Medicines in Low- and Middle-Income Countries: A Scoping Review*, 9 The Lancet regional health Europe 100,219 (2021). https://doi.org/10.1016/j.lanepe.2021.100219

88 Wells, J. C., et al., *An Analysis of Contemporary Oncology Randomized Clinical Trials From Low/Middle-Income vs High-Income Countries*, 7 Jama oncology 379–385 (2021). https://doi.org/10.1001/jamaoncol.2020.7478

89 UN General Assembly, Charter of Economic Rights and Duties of States, A/RES/3281, UN General Assembly, 6 November 1974, https://www.refworld.org/legal/resolution/unga/1974/en/20519 [accessed Nov. 18 , 2024].

90 UN General Assembly, Charter of Economic Rights and Duties of States, A/RES/3281, UN General Assembly, 6 November 1974, https://www.refworld.org/legal/resolution/unga/1974/en/20519 [accessed Jan. 28, 2025].

91 Brower, C. N., and TEPE J, *The Charter of Economic Rights and Duties of States: A Reflection or Rejection of International Law?*, 9 The International Lawyer 295–318 (1975). http://www.jstor.org/stable/40704931

92 Susan Sell, *Intellectual Property and Public Policy in Historical Perspective: Contestation and Settlemen*t, 38 Loy. L.A. L. Rev. 267 (2004). Available at: https://digitalcommons.lmu.edu/llr/vol38/iss1/6

93 Edward S Dove, *Confidentiality, Public Interest, and the Human Right to Science: When Can Confidential Information Be Used for the Benefit of the Wider Community?*, 10 Journal of Law and the Biosciences lsad013 (2023), https://doi.org/10.1093/jlb/lsad013

94 Intellectual property rights and human rights, UN. Subcommission on the Promotion and Protection of Human Rights (52nd sess.: 2000: Geneva). E/CN.4/Sub.2/RES/2000/7.

95 Geneva Declaration on the Future of the World Intellectual Property Organization. October 2004. Available on: http://www.cptech.org/ip/wipo/futureofwipodeclaration.html

96 Fruzsina Molnár-Gábor, *Implementing the Human Right to Science in the Context of Health-Related Data Processing*, 11 Journal of Law and the Biosciences lsae004 (2024), https://doi.org/10.1093/jlb/lsae004

97 Directive 96/9/EC of the European Parliament and of the Council of 11 March 1996 on the legal protection of databases. OJ L 77, 27.3.1996, p. 20–28.

98 WIPO Copyright Treaty art. 10, Dec. 20, 1996, Stat. 112:2860, U.N.T.S. 2186:152. TRIPS Agreement.

99 Anna Nuechterlein et al., *Open Science in Play and in Tension with Patent Protections,* 10 Journal of Law and the Biosciences lsad016 (2023), https://doi.org/10.1093/jlb/lsad016

100 GA Zakout *Public versus Private Interests in Intellectual Property Rights Law: Where does the Right to Science Stand in Cancer Research?*, 8 Eur Health and Pharmaceutical Law Rev 3–17 (2025). https://doi.org/10.21552/ehpl/2025/1/4

101
Moyn, S. Not Enough: Human Rights in an Unequal World. Harvard University Press (2018). https://doi.org/10.2307/j.ctv24trcgs

102 The Lancet Regional Health-Western Pacific, *The Intersection of Science and Governance*, 27 The Lancet Regional Health Western Pacific 100,625 (2022). https://doi.org/10.1016/j.lanwpc.2022.100625

103 Martha Kinsella and Jacob Carter. *State and Local Governments Across the Country Are Sidelining Science*. November 10, 2023. Stat News. Available on: https://www.statnews.com/2023/11/10/state-local-government-science-health-data-politicization-whistleblowers/

104 Widespread Abuse of Science in State Governments Demands Action. Union of Concerned Scientists. Published Sep. 27, 2023. Available on: https://www.ucsusa.org/about/news/widespread-abuse-science-state-governments-demands-action

105 Druckman, J. N., *Threats to Science: Politicization,* 700 *Misinformation, and Inequalities*. The Annals of the American Academy of Political and Social Science 8–24 (2022). https://doi.org/10.1177/00027162221095431

106 Rovelli, C, *Politics Should Listen to Science, Not Hide Behind It*. Nat. Mater. 20, 272 (2021). https://doi.org/10.1038/s41563-020-00891-3

107
Fleck, Leonard M., Precision Medicine and Distributive Justice: Wicked Problems for Democratic Deliberation (New York, 2022; online edn, Oxford Academic, 22 Sept. 2022), https://doi.org/10.1093/oso/9780197647721.001.0001, accessed Nov. 15, 2024.

108 Henderson RH, et al. *Delivering the Precision Oncology Paradigm: Reduced R&D Costs and Greater Return on Investment through a Companion Diagnostic Informed Precision Oncology Medicines Approach*, 16 J Pharm Policy Pract 84 (2023). http://dx.doi.org/10.1186/s40545-023-00590-9.

109 Calvin W L Ho, *Implementing the Human Right to Science in the Regulatory Governance of Artificial Intelligence in Healthcare*, 10 Journal of Law and the Biosciences lsad026 (2023), https://doi.org/10.1093/jlb/lsad026

110 Negri S, *The European Union as a Global Health Actor: Challenges and Opportunities*, 52 Journal of Law, Medicine & Ethics 755–758 (2024). http://dx.doi.org/10.1017/jme.2024.146

111 Consolidated Version of the Treaty on the Functioning of the European Union - Part Three: Union Policies And Internal Actions - Title Vii: Common Rules On Competition, Taxation And Approximation Of Laws - Chapter 3: Approximation of laws - Article 114 (ex Article 95 TEC).

112 Henderson RH, et al., *Spend Less to Achieve More: Economic Analysis of Intermittent versus Continuous Cetuximab in KRAS Wild-Type Patients with Metastatic Colorectal Cancer*, 33 J Cancer Policy 100,342, 2022. http://dx.doi.org/10.1016/j.jcpo.2022.100342.

113 S Sean Tu et al., *Accelerating Biosimilar Market Access: The Case for Allowing Earlier Standing*, 12 Journal of Law and the Biosciences lsae030 (2025), https://doi.org/10.1093/jlb/lsae030

114 Andrea Boggio, A*nticipation in the Biosciences and the Human Right to Science*, 11 Journal of Law and the Biosciences, lsae002 (2024), https://doi.org/10.1093/jlb/lsae002

115 McHenry L, *Biomedical Research and Corporate Interests: A Question of Academic Freedom*, 6 Mens Sana Monogr 146–56 (2008). http://dx.doi.org/10.4103/0973-1229.37086.

116 Ahmed A, *A Critique of Expertise for Health Law*, 50 Journal of Law, Medicine & Ethics 682–686 (2022). http://dx.doi.org/10.1017/jme.2023.8

117 Martin Enserink, *New Dutch Right-Wing Coalition to Cut Research, Innovation, and Environmental Protections*, Science (2024). http://dx.doi.org/10.1126/science.zewr61j

118 Richard L. Hudson. *Assessing the Trump factor in US science: fear and uncertainty*. 07 Nov 2024. Available on: https://sciencebusiness.net/news/international-news/assessing-trump-factor-us-science-fear-and-uncertainty

119 Jeffrey Mervis, *Amid the uncertainty, here’s what Trump’s victory might mean for U.S. science*. Science Insider. 6 Nov 2024. Available on: https://www.science.org/content/article/amid-uncertainty-here-s-what-trump-s-victory-might-mean-u-s-science

120 Mitch Ambrose, *Science Policy Outlook for the Second Trump Presidency*. Nov 14, 2024. Available on: https://ww2.aip.org/fyi/science-policy-outlook-for-the-second-trump-presidency

121 Volker Türk, *Protect the ‘right to science’ for people and the planet*. 623 Nature 9 (2023). https://doi.org/10.1038/d41586-023-03332-8.

